# Modulating silver diamine fluoride chemical derivatives by solid-binding peptides

**DOI:** 10.3389/fbioe.2026.1896633

**Published:** 2026-08-10

**Authors:** Erhan Demirel, Malcolm L. Snead, Paulette Spencer, Candan Tamerler

**Affiliations:** 1 Institute for Bioengineering Research, University of Kansas, Lawrence, KS, United States; 2 Center for Craniofacial Molecular Biology, Herman Ostrow School of Dentistry of USC, The University of Southern California, Los Angeles, CA, United States; 3 Department of Mechanical Engineering, University of Kansas, Lawrence, KS, United States; 4 Bioengineering Program, University of Kansas, Lawrence, KS, United States

**Keywords:** biofunctionalization, biomimetic engineering, caries, heterogeneous tissue interface, peptide modulator, Raman spectroscopy, SDF, X-ray photoelectron spectroscopy

## Abstract

Peptide modulators offer a means to transition from passive dental treatment to actively designing the biological interface with specific responses for efficacy and minimal off-target effects. The oral cavity is exposed to one of the most hostile and dynamic environments in the human body. Targeted delivery of peptide modulators may direct the desired biological outcomes at the interface of dental bioceramic tissues. While noninvasive silver diamine fluoride (SDF) has revolutionized cavity management in early childhood caries, its universal use is limited by its drawbacks including permanent black stain on treated tissues. To mitigate these drawbacks, we investigated the effects of a silver diamine fluoride modulating peptide (SDF-ModP) on silver compound formation and staining outcomes in SDF-treated dentin. Slabs of human dentin tissue were treated with SDF alone or SDF followed by SDF-ModP. Raman micro-spectroscopy and X-ray photoelectron spectroscopy (XPS) were used to identify the silver compounds. The color change was monitored over time using the CIELAB color space. Raman analysis identified silver chloride (AgCl), silver (I) oxide (Ag_2_O), and silver (II) oxide (AgO) as the most abundant compounds formed in SDF-treated dentin. XPS deconvolution of the Ag 3*d*_5/2_ region confirmed the coexistence of metallic silver (Ag (0)), Ag (I), and Ag (III) species. SDF-ModP treatment modulated the distribution of these species, reducing both metallic silver and Ag (III) oxide formation, while increasing the proportion of Ag (I) compounds. Over a 7-day monitoring period, time-resolved color analysis revealed that SDF-ModP delayed and reduced overall black staining compared to SDF alone. The peptide modulated silver chemistry by reducing the formation rate of metallic silver chromophores and higher-valence oxides, thereby decreasing dentin discoloration. These findings suggest that SDF-modulating peptides can be used to improve the therapeutic efficacy and esthetic outcomes of SDF therapy. Our approach represents a molecular biomimetic engineering strategy in which specific peptide interactions with metal ions to modulate the biological interface. This strategy could be extended to other metal-based therapeutics, where precise control of metal ion speciation is desired.

## 1 Introduction

Dental caries is the most prevalent infectious disease of mankind, posing a significant health issue globally (Kassebaum et al., 2015; Pitts et al., 2021; Qin et al., 2022; Sun et al., 2024). Despite advances in prevention and treatment, there is a continuing need for accessible, minimally invasive therapies that can arrest the carious lesions and prevent further decay. This shift in focus has moved the field from invasive restorations towards topical strategies that target early carious lesions (Gao et al., 2016; Caprioglio et al., 2018). Silver diamine fluoride (SDF) has emerged as a leading noninvasive caries-arresting agent owing to its potent antibacterial activity and ease of application (Zheng et al., 2022). SDF has gained widespread acceptance owing to its low cost, ease of use, speed of application, and clinical effectiveness and safety. However, irreversible black staining of treated lesions limits their wider adoption of SDF owing to esthetic limitations (Peng et al., 2012; Crystal and Niederman, 2019). The mechanism(s) underlying staining, particularly within heterogeneous carious tissues, remain poorly understood, and effective mitigation strategies have not yet been developed.

SDF delivers both silver and fluoride ions, and both components react with the mineral and organic elements of the teeth. The exact antimicrobial mechanism has not been identified, but silver ions are known to penetrate carious lesions to provide antibacterial activity by disrupting metabolism and biofilm formation, whereas fluoride ions promote remineralization through the formation of calcium fluoride and fluorapatite deposits (Oliveira et al., 2019). SDF application induces the formation of multiple silver compounds, including Ag_3_PO_4_ (Shah et al., 2018; Li et al., 2019), Ag_2_S (Mei et al., 2018; Li et al., 2019; Toopchi et al., 2021), AgCl (Mei et al., 2013; Mei et al., 2017; Mei et al., 2018), metallic silver (Mei et al., 2013; Mei et al., 2018; Li et al., 2019; Sayed et al., 2020; Hunwin et al., 2024), and silver oxides (Li et al., 2019; Sayed et al., 2020; Srisomboon et al., 2021; Hunwin et al., 2024), each contributing to different efficacies. However, some of these compounds also contribute to the permanent black staining. The fundamental challenge in addressing SDF’s black stain formation lies in the incomplete understanding of silver-tissue interactions at the molecular level within the complex, heterogeneous environment of carious dentin. Although SDF treatment results in the formation of multiple silver and fluoride compounds, the precise mechanisms governing the preferential formation of these compounds, their spatial distribution within the lesion, and their temporal evolution remain poorly characterized (Mei et al., 2018; Li et al., 2019; Kaur et al., 2024). The carious environment presents another unique chemical landscape with variations in pH, protein content, mineral density, and bacterial species across different regions of the lesion (Simon-Soro et al., 2013; Kianoush et al., 2014). Yet, the influence of these microenvironmental factors on silver and fluoride speciation and compound stability is not well understood. This knowledge gap has significant implications because different silver compounds exhibit distinct optical properties, antimicrobial activities, and long-term stability profiles (Zhao et al., 2018). Without a comprehensive understanding of the factors that control silver compound formation and distribution, the development of targeted strategies to selectively promote beneficial compounds while minimizing those responsible for discoloration remains empirical and limited in scope.

To overcome the esthetic drawbacks of SDF, other silver-based strategies, such as silver nitrate and silver nanoparticles, and stain-mitigating approaches, such as AgF/KI and AgF/SnF_2_, have been explored (Xu et al., 2024). Silver nanoparticles have demonstrated compelling bactericidal activity against *Streptococcus mutans* by penetrating the bacterial cell wall and disrupting intracellular targets, with some studies reporting an order-of-magnitude greater antibacterial efficacy than conventional agents like chlorhexidine (Khubchandani et al., 2022; Karaduran et al., 2024). Furthermore, combination formulations that modulate silver’s reactivity to reduce staining, such as sequential treatment protocols involving AgF/KI and AgNO_3_ followed by fluoride varnish, have been clinically explored, despite concerns regarding the survival rate of restorative materials following KI application and the compromise of antibacterial activity (Duangthip et al., 2017; Roberts et al., 2020; Al-Hijazi et al., 2023; Horst et al., 2023). Other studies have investigated alternative substances to address the staining issue, such as glutathione (GSH) (Sayed et al., 2018) and different chemical formulations, such as nano-silver fluoride (NSF) (Espindola-Castro et al., 2020) and polyethylene glycol (PEG)-coated nanoparticles containing sodium fluoride (NaF) (Zhao et al., 2020). Although these strategies are promising, they are limited by inconsistent results and an incomplete understanding of silver chemistry in complex biological tissues.

The limitations of current chemical approaches to modulate silver chemistry and black stain formation have prompted us to explore molecular biomimetic strategies including peptide-modulators, which offer molecular-level control for selectively interacting with and altering the function of diverse biological targets ranging from proteins to mineralized tissues (Souroujon and Mochly-Rosen, 1998; Warotayanont et al., 2008; Lee et al., 2010; Gungormus et al., 2012; Zhou et al., 2015; Cunningham et al., 2017; Woolfolk et al., 2022). Several peptide modulators have been identified with the mechanism of action for orthosteric and allosteric modulation that target intrinsically disordered domains and for modulating nucleation modulation when modulating nucleation, growth and morphology of biological composites (Tamerler and Sarikaya, 2006; Lee et al., 2010; Gungormus et al., 2012; Olson et al., 2021; Mannes et al., 2022). We and other groups have published on solid-binding peptides serving as molecular adaptors at inorganic-biological interfaces, through sequence-encoded combinations of polar-, charged-, and aromatic-residues to achieve material-specific recognition and binding (Tamerler et al., 2003; Oren et al., 2005; Tamerler and Sarikaya, 2007; Heinz et al., 2009; Tamerler and Sarikaya, 2009; Wen et al., 2011; Chiu et al., 2012; Hnilova et al., 2012a; Zhou et al., 2015; Hellner et al., 2019; Walsh and Knecht, 2019; Xie et al., 2019; Ruan et al., 2022; Cloyd et al., 2023; Perdomo et al., 2023; Mao et al., 2024). Beyond binding affinity, these peptides can modulate the nucleation, growth, and redox behavior of the target inorganic phase, enabling applications ranging from remineralization to nanomaterials and biohybrid interface design (Hnilova et al., 2012a; Hnilova et al., 2012b; Xie et al., 2019; Cloyd et al., 2023). Our group has used combinatorial, computational, and machine learning tools to design and tune solid-binding peptides with a selective affinity for several inorganic materials, including silver (Tamerler et al., 2003; Tamerler and Sarikaya, 2007; 2009; Hnilova et al., 2012a; Boone et al., 2021; Cloyd et al., 2023). The peptide SDF-ModP (EQLGVRKELRGV) demonstrates strong silver-binding characteristics and influences the formation of silver compounds (Hnilova et al., 2012c; Sedlak et al., 2012). In a previous study, we engineered a bifunctional peptide that integrated SDF-ModP with an amelogenin-derived sequence to facilitate the remineralization of SDF-treated dentin (Woolfolk et al., 2022).

In the present study, we investigated how SDF-modulating peptide (SDF-ModP) interacts with SDF. The peptide modulatory properties were explored for the formation of chemical compounds that contribute to clinically desired outcomes and those contributing to esthetic stains that hamper the wider therapeutic application of SDF. Here, we applied Raman microspectroscopy and X-ray photoelectron spectroscopy (XPS) analyses to characterize the SDF-dentin interface to identify silver compounds formed in the presence and absence of SDF-ModP peptide treatment, while changes to the black stain were analyzed using CIELAB color space measurements. Our results show the promise of the SDF-modulating peptide biomimetic pathway to enhance the clinical utility of SDF treatment while modulating SDF’s drawbacks.

## 2 Materials and methods

### 2.1 Materials

Sodium chloride (NaCl), sodium bicarbonate (NaHCO_3_), potassium chloride (KCl), potassium phosphate dibasic trihydrate (K_2_HPO_4_·3H_2_O), magnesium chloride hexahydrate (MgCl_2_·6H_2_O), hydrochloric acid (HCl), calcium chloride (CaCl_2_), sodium sulfate (Na_2_SO_4_), tris (hydroxymethyl) aminomethane, phosphoric acid (85 wt% in H_2_O), dimethylformamide (DMF, 99.8%), ethanol (≥99.45%, 200 proof), trifluoroacetic acid (TFA, 99%), triisopropylsilane (98%) and hydroxyapatite (HAp, Ca_5_(OH) (PO_4_)_3_, nano-powder, <200 nm particle size (BET), ≥97%, synthetic) were sourced from Sigma-Aldrich Inc., (St. Louis, MO, United States). Acetonitrile (Optima LC/MS Grade) and phenol (89%) were supplied by Fisher Scientific Company LLC (Pittsburgh, PA, United States). A 38% silver diamine fluoride (SDF) solution was obtained from Dengen Dental (Bahadurgarh, Haryana, India). All other materials were acquired from Sigma-Aldrich (St. Louis, MO, United States). All the materials were used as received without further purification.

### 2.2 Peptide synthesis

The SDF-modulating peptide (SDF-ModP; sequence: EQLGVRKELRGV) was synthesized using an Fmoc-based solid-phase peptide synthesis protocol on an AAPPTEC Focus XC synthesizer (AAPPTEC, Louisville, KY, United States). After synthesis, the mixture was washed with dimethylformamide (DMF) and 200-proof ethanol and dried under vacuum at room temperature. The peptide was cleaved from the resin, and the side chains were deprotected following the standard AAPPTEC protocol using a cleavage cocktail containing trifluoroacetic acid (TFA), phenol, triisopropylsilane, and deionized water at a ratio of 90.0:5.0:2.5:2.5 (v/v). Peptide cleavage was executed for three hours on a rotator. The peptide was precipitated with cold ether and then lyophilized. An analytical HPLC system, LC-2010 HT liquid chromatograph (Shimadzu Scientific Instruments, Columbia, MD, United States), and SPD-M20A prominence diode array detector (Shimadzu Scientific Instruments, Columbia, MD, United States) were used to confirm peptide purity. A 5 μm C-18 silica Luna column (250 mm × 4.6 mm, Phenomenex Inc., Torrance, CA, United States) was used with a mobile phase consisting of 100% acetonitrile and 99.9% HPLC-grade water containing 0.1% trifluoroacetic acid (TFA). The system was operated with a linear gradient at a flow rate of 1 mL/min, and the detection wavelength was 254 nm. The lyophilized peptide was stored at −20 °C until use in subsequent experiments. The purity of SDF-ModP was determined to be 91.88% by HPLC, and the chromatogram is shown in Supplementary Figure S1.

### 2.3 Dentin tissue sample preparation

The specimens used in this study consisted of human teeth scheduled for extraction as part of the therapeutic treatment plan. These teeth would otherwise be discarded, and they are not associated with patient identifiers to ensure anonymity. Consequently, this study did not meet the definition of human subject research, as confirmed by the Institutional Review Board of the University of Kansas Human Research Program. After extraction, the teeth were cleaned, placed in individual vials filled with phosphate-buffered saline solution modified with 20 ppm sodium azide, and stored at +4 °C.

**FIGURE 1:**
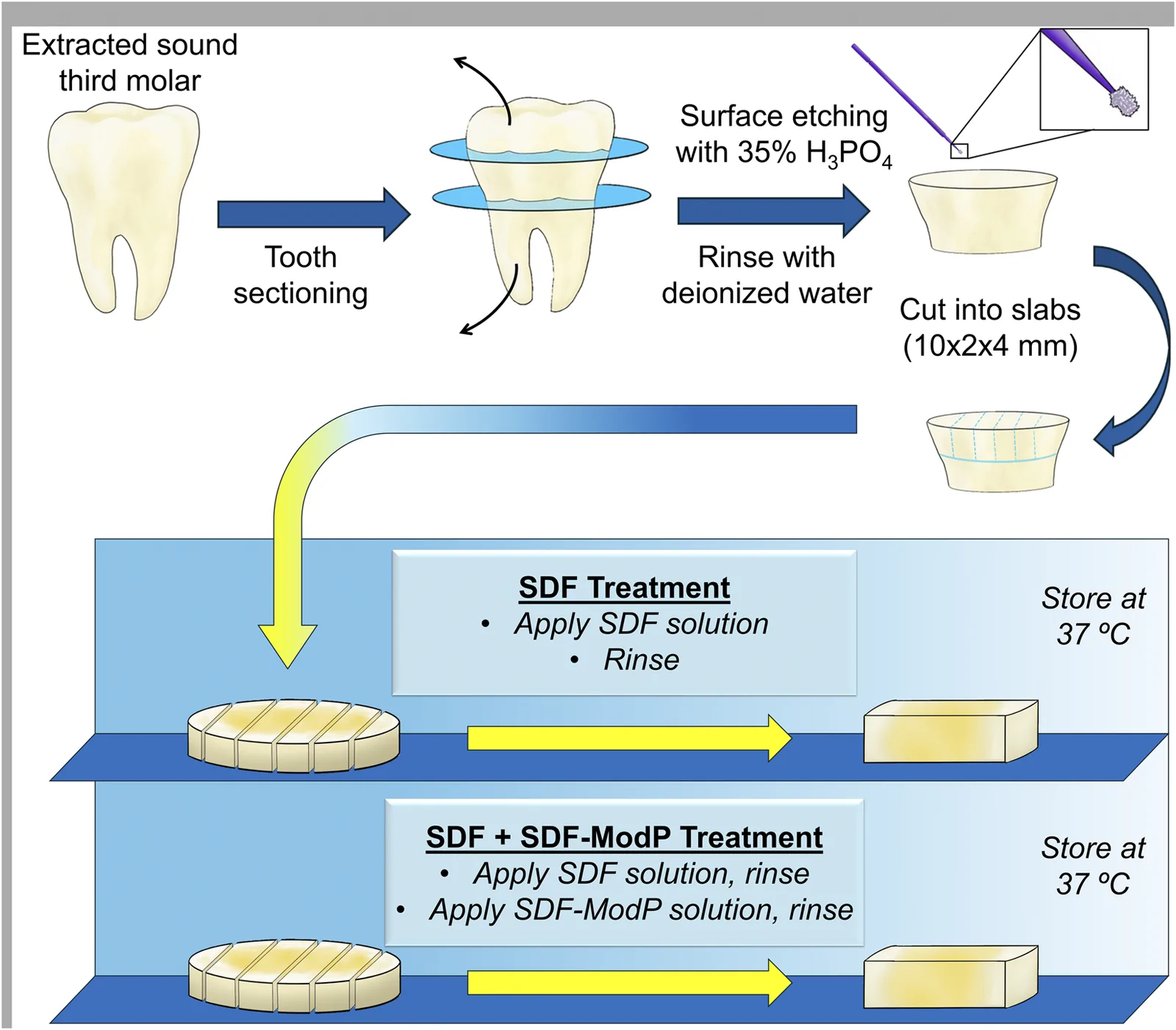
Schematic illustration of the procedure for preparing human dentin slabs for SDF and SDF-ModP applications.

The occlusal one-third of the crown was sectioned perpendicular to the long axis of the tooth using a water-cooled low-speed diamond saw (Buehler Ltd., Lake Bluff, IL, United States). A uniform smear layer was created by abrading the exposed dentin surface with 600-grit silicon carbide in water. The exposed dentin surfaces were demineralized with 35% phosphoric acid for 60-s, and then rinsed with deionized water. This demineralization step was used to generate a standardized, reproducible reactive dentin surface for chemical analysis rather than to model a natural carious lesion. Etching the dentin with 35% phosphoric acid is a well-established method for modifying the smear layer and exposing the collagen-mineral substrate (Spencer et al., 2001; Spencer et al., 2005; Wang et al., 2006; Wang et al., 2007). This well-established acid-etching method was used here to reduce inter-sample variability in mineral content prior to SDF treatment. All dentin tissue slabs, including the untreated controls, underwent identical demineralization and storage conditions prior to group-specific treatment. This approach was used to ensure that the observed differences between the groups reflected the treatment effects rather than baseline tissue variability. The diamond saw was used to make parallel cuts spaced 2 mm apart, perpendicular to the dentin surface, and dentin tissue slabs were separated by cutting 4 mm below their surface. The resulting dentin tissue slabs were approximately 8–10 mm long, 2 mm wide, and 4 mm thick (Figure 1) (Spencer et al., 2005; Wang et al., 2006; Wang et al., 2007). The ISO 23317:2014 standard was followed, and dentin tissue slabs were stored at +4 °C in simulated body fluid (SBF, pH 7.4) to replicate mammalian saliva conditions (SBF composition shown in Supplementary Table S1) (Oyane et al., 2003; ISO, 2014; Srisomboon et al., 2022).

Dentin tissue slabs were prepared from multiple extracted human third molars, all clinically healthy and processed under identical preparation and storage conditions (Section 2.3). The slabs were pooled and randomly distributed across the treatment groups for each analytical method. Raman micro-spectroscopy used 15 slabs (5 per group) sourced from 3 M, and XPS and chromatic difference tests used nine slabs (three per group) sourced from 2 M for each method. This pooling-and-randomization approach was used to distribute any inter-tooth variability evenly across the treatment groups rather than confounding it with the treatment effect.

### 2.4 SDF and SDF-ModP applications

The guidelines of the University of California, San Francisco (UCSF) Caries Arrest Committee and the manufacturer were followed to apply SDF to dentin tissue slabs (Horst, 2018; Horst et al., 2023). Briefly, the dentin tissue slabs were removed from the SBF, rinsed once with deionized water, and dried with compressed air to prevent SDF dilution and expose the dentin tissue. SDF was transferred to a fresh plastic dish, covered to protect it from light, and applied to the human dentin slab for 1-min before rinsing with deionized water. To mimic the oral environment, the treated tissue samples were placed individually in the modified SBF solution and incubated at 37 °C for 6-h.

Lyophilized SDF-ModP peptide stocks were reconstituted in deionized water to a concentration of 50 μM. The dentin slabs were rinsed with deionized water and gently dried with compressed air. The peptide solution was drop-cast onto the dentin slabs, incubated for 2-h at room temperature in the dark, and excess peptide solution was removed by gentle rinsing with deionized water. The tissue slabs were stored in a fresh SBF solution until they were analyzed.

### 2.5 Raman microspectroscopy analysis

To evaluate the chemical changes induced by SDF treatment and their modification by the SDF-ModP peptide, two sets of samples were prepared for Raman microspectroscopy. First, a model experiment was conducted using synthetic hydroxyapatite (HAp) powder, which was pressed into disks. Three groups were established: untreated HAp, HAp treated with SDF and incubated in deionized water, and HAp treated with SDF and incubated in SBF. In parallel, human dentin tissue slabs were divided into three groups: (i) untreated, (ii) SDF, and (iii) SDF followed by SDF-ModP application.

Hydroxyapatite (HAp) disks (n = 5) and human dentin tissue slabs (n = 5) were mounted on glass slides and analyzed using a LabRAM ARAMIS Raman microscope (HORIBA Jobin Yvon, Edison, NJ, United States), equipped with a 785 nm diode laser (LD current: 200 mA, Sacher Lasertechnik, PilotPC 500, Marburg, Germany) using a motorized stage (Märzhäuser Wetzlar GmbH & Co. KG, Wetzlar, Germany). Ten Raman spectra were acquired over a wavelength range of 200–1,200 cm^−1^ with an acquisition time of 10-s using a ×50 objective for HAp disks and a ×60 water immersion objective for dentin tissue slabs. The resulting scattered light was collected using a Synapse CCD (HORIBA Scientific, Edison, NJ, United States) cooled to −70 °C. The instrument was set to a 100 μm slit, 150 μm confocal hole, and 600 gr/mm grating. The data were processed using the LabSPEC 6 software (version 6.7.1.10, HORIBA Scientific, Edison, NJ, United States) and normalized according to the most intense peak.

### 2.6 X-ray photoelectron spectroscopy (XPS)

X-ray photoelectron spectroscopy (XPS) analysis was performed on non-treated human dentin tissue slabs, SDF-treated human dentin tissue slabs, SDF+SDF-ModP-treated human dentin tissue slabs, and SDF-treated HAp exposed to light (550 mW/cm^2^ for 10-s using a commercial visible-light curing unit, Spectrum 800, Dentsply, Charlotte, NC, United States) (n = 3 independent HAp disks or human dentin slabs per treatment group), using a PHI 5000 Versa Probe II instrument (ULVAC-PHI Inc., Kanagawa, Japan) under ultrahigh vacuum conditions (1 × 10^−9^ bar). The X-ray source utilized was Al–Kα (λ = 1,486.6 eV) with a power of 25 W for survey scans and 47 W for high-resolution scans, using a beam diameter of 100 µm. Wide-scan survey spectra were acquired over a binding energy (BE) range of 0–1,100 eV to determine the chemical elemental composition with an energy step of 1.0 eV. High-resolution detailed scans were obtained at an energy step of 0.1 eV. Detailed scans of the chemical element orbits for C 1*s*, O 1*s*, N 1*s*, P 2*p*, Ca 2*p*, F 1*s*, Ag 3*d*, Cl 2*p*, and Na 1*s* were performed. CasaXPS software (Version 2.3.26PR1.0, Casa Software Ltd., Teignmouth, United Kingdom) was used to analyze the XPS data, and all spectra were calibrated using the C 1*s* peak at 284.6 eV.

### 2.7 Chromatic difference analysis

Images of untreated control, SDF-treated, and SDF+SDF-ModP-treated human dentin tissue slabs (n = 5 independent samples per treatment group) were captured using a Nikon D300 camera equipped with a Nikon AF-S Micro Nikkor 105 mm f/2.8G ED lens (Nikon, Melville, NY, United States) illuminated by two soft box studio lights with diffusers (B&H, NY, United States). The camera settings were ISO 400, aperture f/40, and shutter speed 1/15-s in manual mode. Images were captured at baseline (time 0) and at regular intervals of 6, 12, 24, 36, 48, 72, 96, 120, 144, and 168-h after application. Images were processed using Fiji software (ImageJ, Version 1.54p). The white balance was adjusted for each image using the IJP White Balance plugin with a white balance calibration chart as a reference, and the images were converted into L*a*b* stacks. The mean gray value was determined from the control dentin tissue slabs, and the color difference (ΔE) relative to the baseline image was calculated based on the CIELAB space with the parametric weighting factors *k*_*L*_ *, k*_*C*_ *,* and *k*_*H*_, according to the following equation: The remaining color difference was computed following the approach described by Sharma et al. (2005).

$$\Delta E_{00}\left(L_{1}^{*},a_{1}^{*},b_{1}^{*};L_{2}^{*},a_{2}^{*},b_{2}^{*}\right)=\sqrt{{\left(\frac{\Delta L^{\prime }}{k_{L}S_{L}}\right)}^{2}+{\left(\frac{\Delta C^{\prime }}{k_{C}S_{C}}\right)}^{2}+{\left(\frac{\Delta H^{\prime }}{k_{H}S_{H}}\right)}^{2}{+R}_{T}\left(\frac{\Delta C^{\prime }}{k_{C}S_{C}}\right)\left(\frac{\Delta H^{\prime }}{k_{H}S_{H}}\right)}$$

### 2.8 Statistical analysis

Data are presented as mean ± standard error of the mean (±SEM). To assess chromatic differences, one-way analysis of variance (ANOVA) was conducted, followed by Tukey’s *post hoc* test. Data were analyzed using GraphPad Prism (v10.6.1 for Windows, GraphPad Software, Boston, MA, United States), and the resulting p-values were reported.

## 3 Results and discussion

Prior studies have reported the formation of a heterogeneous interface following SDF treatment of carious regions and suggested the involvement of silver ions, formation of silver compounds, and presence of nanostructured silver, such as nanowires and Ag/AgCl nanoparticles, which adversely affect esthetics (Seto et al., 2020; Hunwin et al., 2024). In this study, we conducted a detailed investigation of this interface on human dental tissues to identify the silver compounds and distribution of these compounds following SDF treatment. We also analyzed this interface to determine the chemical outcomes when SDF-modulating peptide is applied after SDF treatment.

### 3.1 Characterization of silver compounds formed by SDF using Raman micro-spectroscopy

**FIGURE 2:**
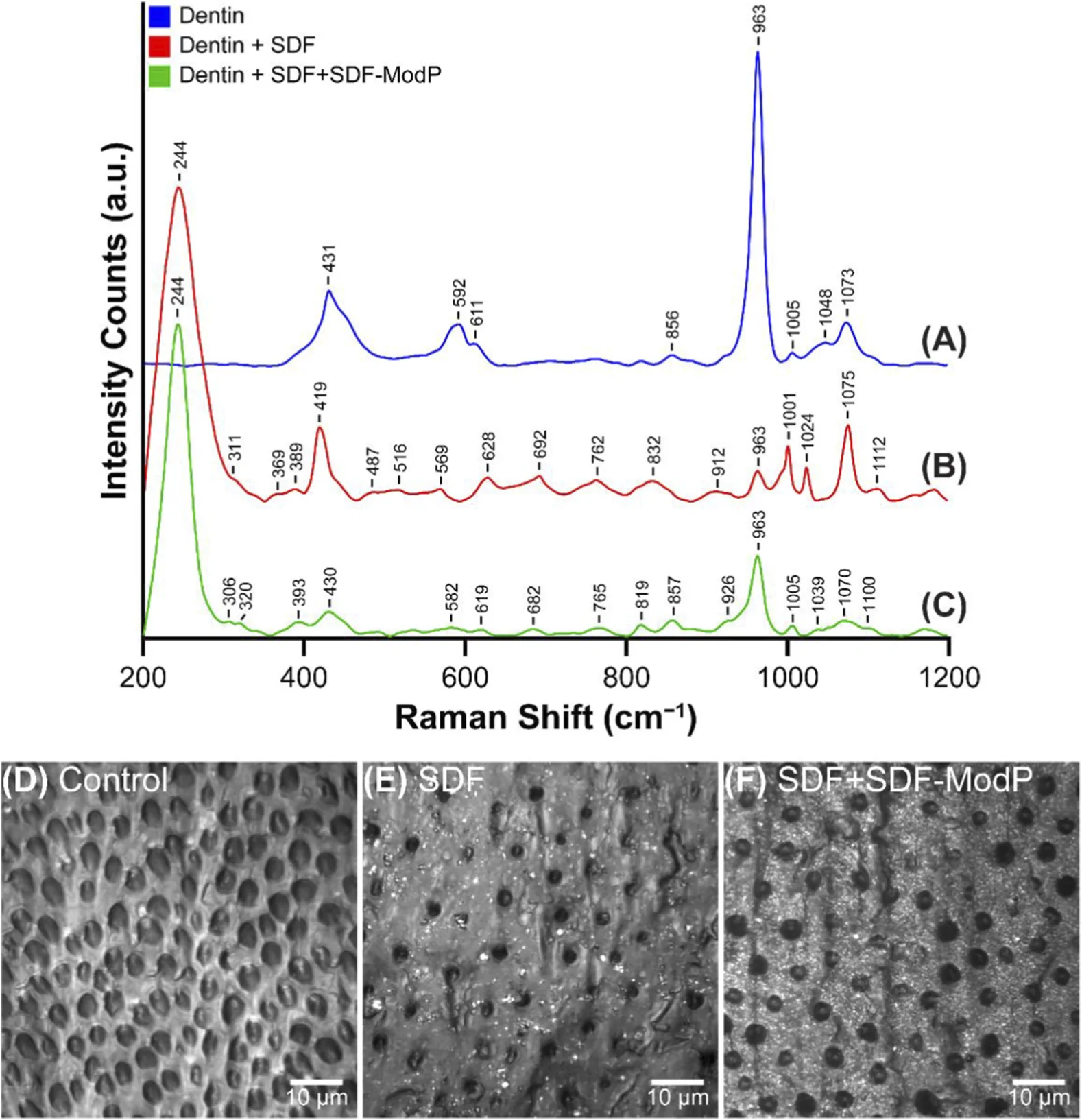
Raman spectra of **(A)** untreated dentin, **(B)** SDF-, and **(C)** SDF+SDF-ModP-treated dentin tissue slabs and confocal bright-field microscopy images of **(D)** untreated, **(E)** SDF- and **(F)** SDF+SDF-ModP-treated dentin surfaces. Scale bars are as shown for each panel (Spectra are representative of 5 independent dentin tissue slabs per treatment group).

Metallic silver is Raman-inactive under visible-light excitation because the atomic vibrations do not cause a change in polarizability. Nonetheless, it is possible to detect silver salts and oxides on human dentin surfaces using their characteristic vibrational signatures. Dentin slabs prepared from human teeth were processed and treated as described in Figure 1 and in the Materials and Methods, Sections 2.3, 2.4. The use of human dentin tissue slabs aimed to examine the treatment effects on a clinically relevant substrate and evaluate the influence of SDF-ModP on their outcomes. Raman spectra of untreated (Figure 2A), SDF treatment alone (Figure 2B), and SDF plus SDF-modulating peptide (SDF-ModP, EQLGVRKELRGV)-treated (Figure 2C) dentin tissue slabs were collected using a 785 nm laser excitation. The spectra were dominated by vibrational modes originating from the mineral and organic components of the dentin tissue. The phosphate signals from hydroxyapatite mineral showed characteristic features at 431 cm^−1^ (*ν*_2_ O-P-O bending mode), 592 and 611 cm^−1^ (*ν*_4_ O-P-O bending mode), and the dominant peak at 963 cm^−1^ corresponding to the symmetric *ν*_1_ P-O band stretching mode, which is the most intense and reliable spectral marker for apatite minerals (Shah, 2020; Mandair et al., 2021). The *ν*_3_ P-O asymmetric stretching mode appeared at 1,048 cm^−1^, and the B-type carbonate substitution in the apatite lattice was evidenced by the peak at 1,073 cm^−1^ (Shah, 2020; Mandair et al., 2021; Unal et al., 2021). The intensity of the P-O stretching bands was more attenuated in the SDF-treated samples than in the untreated dentin samples, suggesting surface modification or possibly masking by silver compounds. Protein spectral peaks characteristic of the collagen-rich organic matrix were identified in all the dentin samples. The peak at 856 cm^−1^ corresponds to proline C-C stretching, while the feature at 1,005 cm^−1^ is assigned to phenylalanine ring breathing vibrations (Ko et al., 2005; Kazanci et al., 2006; Mandair et al., 2021). These amino acid markers serve as internal standards to confirm the preservation of the organic dentin matrix after chemical treatment.

In the SDF-treated dentin samples (Figure 2B), multiple new peaks appeared in the 200–600 cm^−1^ region that were absent in the untreated controls. The most prominent feature at 244 cm^−1^ is assigned to the Ag-Cl stretching mode of silver chloride (AgCl), which forms when silver ions from SDF react with chloride ions present in the simulated body fluid (Waterhouse et al., 2001; Tsendzughul and Ogwu, 2019; Menzel et al., 2023). This assignment is strongly supported by the reference spectra of commercial AgCl (Supplementary Figure S2B) and is consistent with previous reports of AgCl as the primary silver compound in saliva-exposed SDF-treated teeth (Mei et al., 2013; Mei et al., 2018).

Additional peaks in the silver oxide region were identified at 311 cm^−1^, 389 cm^−1^, 487 cm^−1^, and 516 cm^−1^. These features are characteristic of mixed silver-oxide phases. Specifically, peaks at 389 and 487 cm^−1^ correspond to the Ag-O bending and stretching modes of silver (I) oxide (Ag_2_O), while the 311 cm^−1^ peak is diagnostic for silver (II) oxide (AgO, a mixed-valence Ag^+^Ag^3+^O_2_ compound) (Waterhouse et al., 2001; Martina et al., 2012; Tsendzughul and Ogwu, 2019). The formation of both Ag_2_O and AgO is consistent with the reference standard spectra (Supplementary Figures S2C,D) and indicates the partial oxidation of silver species after the treatment process.

The peak at 419 cm^−1^ likely represents an overlap of the dentin phosphate *ν*_2_ mode (431 cm^−1^) with Ag_2_O bulk vibrations (417–430 cm^−1^) (Waterhouse et al., 2001; Shah, 2020), demonstrating the complex interfacial chemistry between silver compounds and the mineral substrate. Novel peaks at approximately 682 cm^−1^ and 762 cm^−1^ were consistently observed in all SDF-treated samples but could not be assigned to known silver compounds or dentin components based on the current literature. These may represent silver-protein complexes or silver-phosphate interaction products, which will be further investigated using complementary techniques.

The SDF+SDF-ModP-treated dentin samples (Figure 2C) showed retention of the AgCl peak at 244 cm^−1^, confirming that silver chloride formation still occurred in the presence of the peptide. However, notable differences were observed in the silver oxide region (300–600 cm^−1^). Peaks attributed to AgO (306 cm^−1^) and Ag_2_O (393 and 430 cm^−1^) were present, but with different relative intensities compared to the SDF-only samples. The modified distribution suggests that SDF-ModP influences the oxidation state equilibrium of the resulting silver species, potentially by complexing with silver ions and modulating their reduction kinetics. The dentin phosphate peaks remained well-resolved in the SDF-ModP-treated samples, with less spectral broadening than that observed in the SDF-only treatment, indicating better preservation of the mineral structure.

Confocal bright-field microscopy images collected during the Raman analysis (Figures 2D–F) provided visual confirmation of the crystal formation patterns. Untreated dentin (Figure 2D) showed dentinal tubules with smooth surfaces and no crystalline deposits. The SDF-treated dentin surface (Figure 2E) showed clear evidence of crystalline deposits, suspected to be water-insoluble silver compounds, including AgCl and silver oxides (Menzel et al., 2023; Kaur et al., 2024), whereas the SDF+SDF-ModP-treated dentin surface (Figure 2F) showed significantly fewer surface deposits, consistent with the peptide’s proposed role in modulating silver compound precipitation at the dentin surface rather than altering the overall amount of silver reaching the tissue.

To further characterize silver compound formation in a simplified system, we analyzed synthetic hydroxyapatite (HAp) disks treated with SDF and incubated them in either deionized water or simulated body fluid (SBF) (Supplementary Figure S3). Untreated HAp showed characteristic phosphate modes at 431, 589, and 962 cm^−1^ (Supplementary Figure S3A), matching the dentin mineral signature. SDF-treated HAp incubated in water (Supplementary Figure S3B) displayed silver oxide features but notably lacked the strong AgCl peak, confirming that chloride from the simulated body fluid contributed to AgCl formation. In contrast, SDF-treated HAp incubated in SBF (Supplementary Figure S3C) showed an intense AgCl peak at 241 cm^−1^, alongside silver oxide signatures, closely replicating the human dentin spectra and supporting the mechanism of chloride-mediated AgCl precipitation under physiologically relevant conditions. Additionally, confocal microscopy confirmed that crystal formation on SDF-treated surfaces was more readily visualized on non-biologic hydroxyapatite substrates than on dentin (Supplementary Figure S4). SDF treatment produced discrete surface precipitates in both water- and SBF-incubated HAp discs (Supplementary Figures S4B,C), consistent with the findings in human dentin samples.

### 3.2 Characterization of silver compounds formed by SDF using X-ray photoelectron spectroscopy (XPS)

The compounds formed upon treatment were thoroughly characterized using X-ray photoelectron spectroscopy (XPS). Untreated dentin, SDF-treated, and SDF+SDF-ModP-treated dentin tissue slabs from healthy human teeth were studied. In parallel with Raman microspectroscopy, we performed further analysis on samples prepared as disks of synthetic hydroxyapatite with SDF treatment. The only difference between these samples was exposure to light irradiation (550 mW/cm^2^) for 10-s to simulate the clinically relevant application of SDF to arrest caries (Supplementary Figures S5, S6).

**FIGURE 3:**
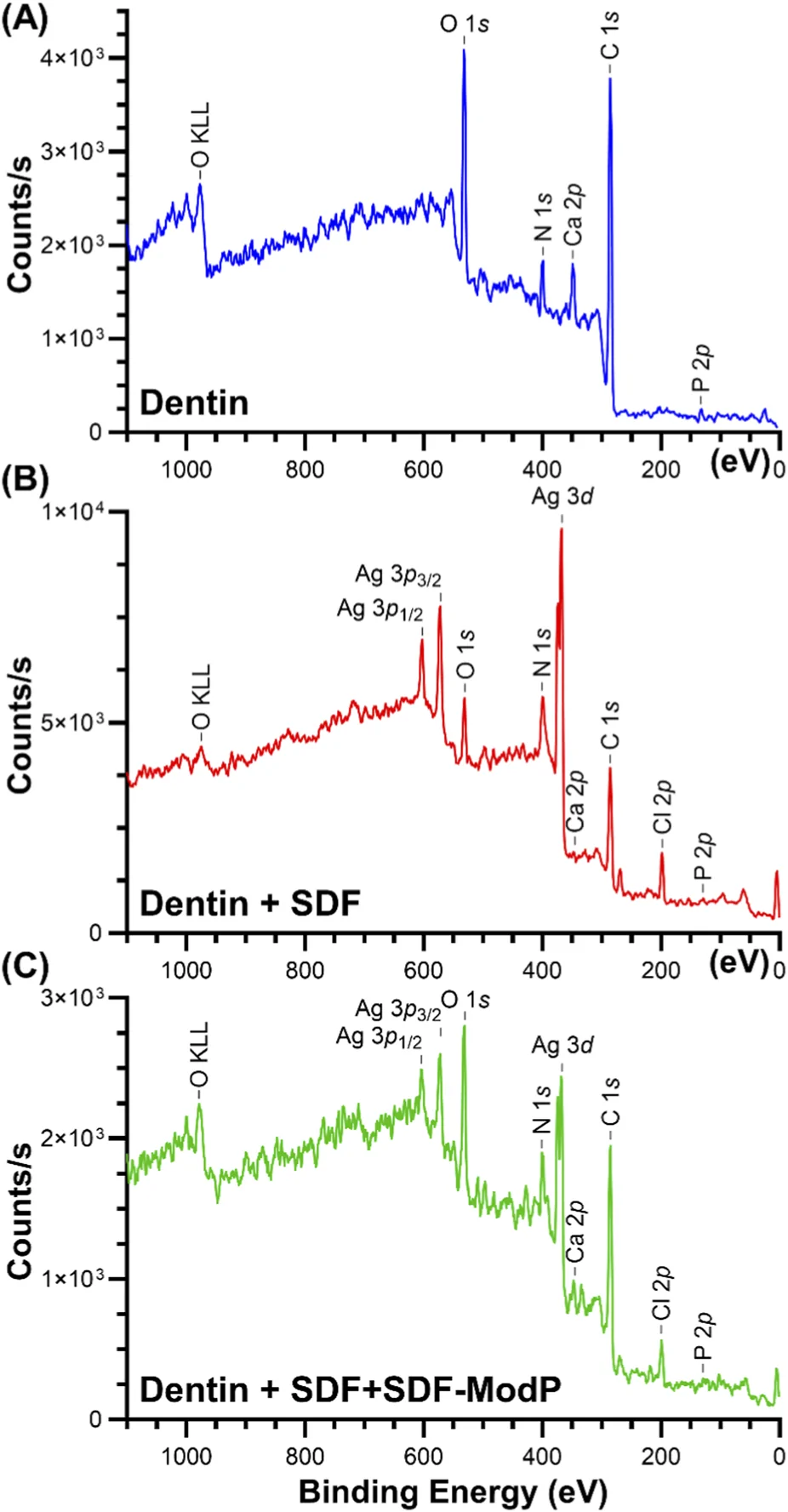
X-ray photoelectron spectroscopy (XPS) survey spectra of human dentin tissue slabs: **(A)** untreated dentin showing characteristic Ca, P, O, N, and C peaks; **(B)** dentin treated with SDF showing additional Ag and Cl signals; and **(C)** human dentin treated with SDF followed by SDF-ModP with similar elemental composition to SDF-only treatment. Key elemental peaks are labeled. Spectra are representative of n = 3 independent samples per treatment group.

A wide-scan survey spectrum (Figure 3) was obtained to determine the elemental composition of the human dentin tissue slabs. Signals originating from Ca, P, O, N, and C were detected in all the samples, whereas Ag and Cl were detected only in the SDF and SDF+SDF-ModP treated samples, respectively. High-resolution scans of the samples were calibrated using the C 1*s* peak at 284.8 eV.

**FIGURE 4:**
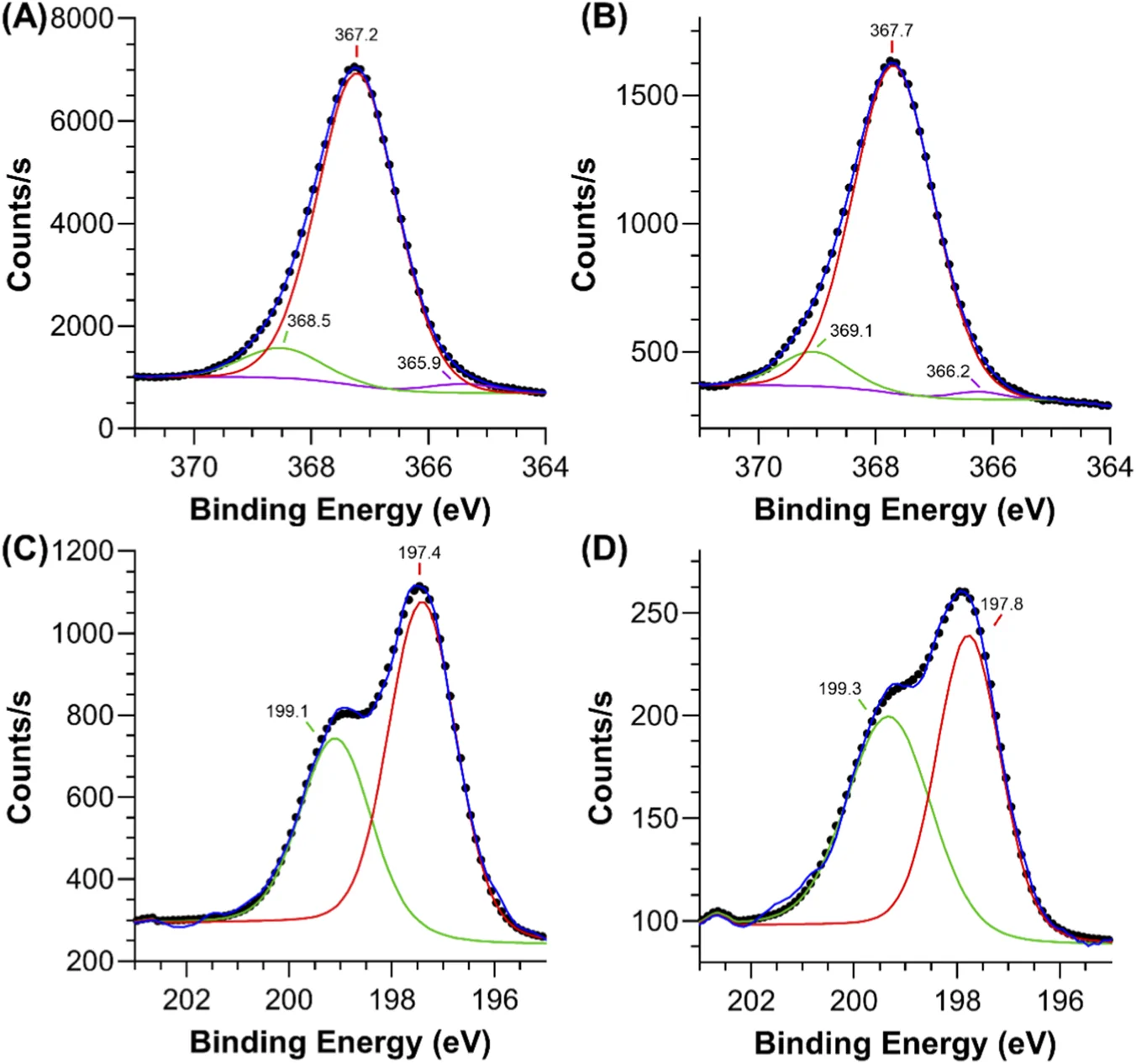
XPS analysis of human dentin tissue slabs treated with SDF alone (left column) and SDF followed by SDF-ModP (right column). High-resolution spectra of the Ag 3*d*_5/2_ region are presented in **(A,B)** for SDF-treated and SDF+SDF-ModP-treated samples, and the O 1*s* regions are presented in **(C,D)**, respectively. Fitted component peaks (Voigt functions) are shown as green, red, and purple lines, with the total fitted envelope in dark blue. Experimentally recorded data points are represented by black dots.

**FIGURE 5:**
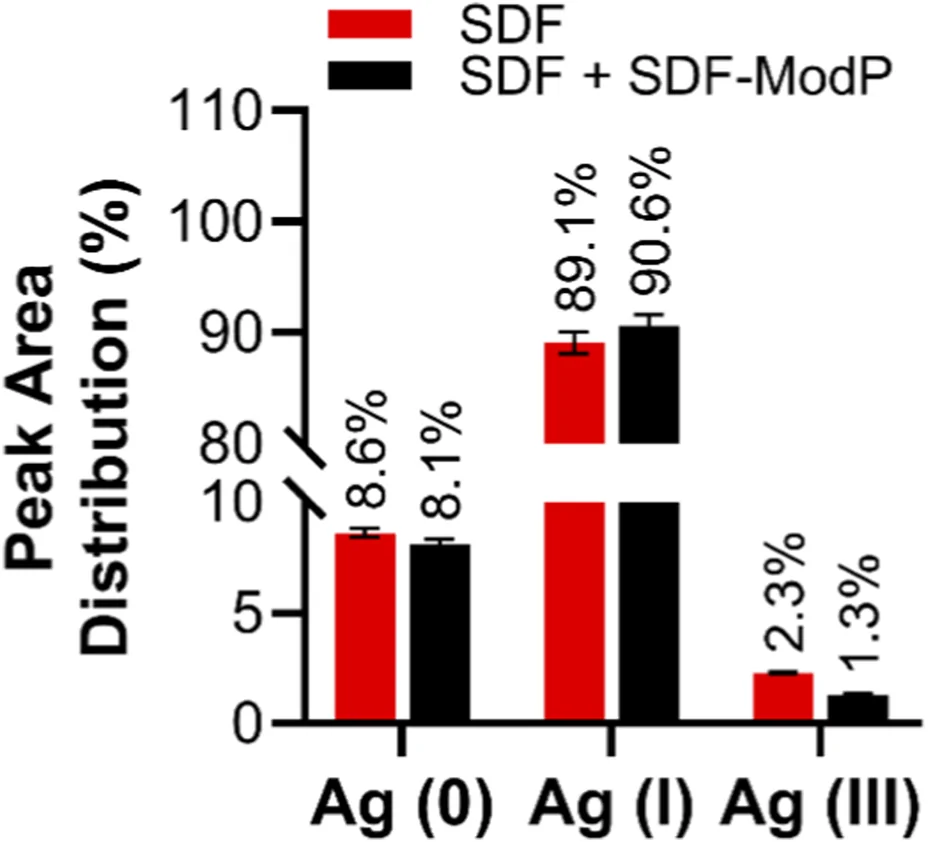
XPS analysis of the Ag 3*d*_5/2_ peak position and peak area distribution of SDF and SDF+SDF-ModP treated samples. Data represents SEM (n = 3 samples per group).

The deconvolution of the Ag 3*d*_5/2_ spectra revealed three peaks for the SDF and SDF+SDF-ModP treated samples (Figure 4). The peaks at 368.5 and 369.1 eV were assigned to metallic Ag, which is consistent with the literature-derived values. The two fitted components within the metallic Ag envelope (368.5 and 369.1 eV for SDF and SDF+SDF-ModP samples, respectively) fall within the range reported in the literature for Ag (0). The small shift between samples is attributed to differences in local charging and surface heterogeneity inherent to a biologically mineralized tissue matrix rather than to two distinct metallic silver species. The peaks at 367.2 and 367.7 eV represent Ag (I), such as in AgCl and Ag_2_O, while 365.9 and 366.2 eV were attributed to the Ag (III) component of the mixed-valence Ag (I,III) oxide (AgO), consistent with prior assignments of this oxidation state in highly oxidized silver films (a mixed-valence Ag^+^Ag^3+^O_2_ compound) (Hoflund et al., 2000; Kaspar et al., 2010; Ferraria et al., 2012; Zhao et al., 2017). On human tooth substrates, silver speciation following SDF treatment is controlled by the chemical elements of the tissue and oral environment. While a portion of Ag^+^ reacts with Cl^−^ to yield AgCl, oxidation and photoreduction of silver species occur over time, and reactions with phosphate-rich surfaces can form Ag_3_PO_4_ before reduction to metallic silver (Mei et al., 2013; Mei et al., 2018; Kaur et al., 2024). These processes support dynamic interconversion between Ag (I)/Ag (III) compounds and Ag (0) on dentin. Although peak integrations revealed similar distributions among the identified silver species, SDF-treated dentin showed slightly higher Ag (0) and Ag (III) contents than SDF+SDF-ModP-treated dentin (Figure 5), suggesting that SDF-ModP treatment influenced the formation of metallic silver and AgO. Several reports have also indicated the involvement of proteins in stabilizing Ag^+^ via nitrogen, oxygen, or sulfur donors, shifting the equilibrium from rapid oxidation or photoreduction to Ag^0^ (Ballottin et al., 2016; Yadav et al., 2023). Perhaps biomineralized tissue, such as human dentin, could chelate Ag^+^ at the collagen-mineral interface, and SDF-ModP may sequester silver ions, reducing their activity and favoring thin, ligand-rich surface complexes with altered final-state outcomes, potentially explaining the observed shifts in the chemical speciation of available Ag^+^ in the SDF-ModP peptide-treated samples. Potential coordination mechanism suggested here would benefit from further studies on binding affinity measurements (ITC, SPR) or structural characterization (CD spectroscopy, molecular docking).

The results obtained from the light-exposed HAp-SDF disk samples revealed a higher intensity of the fitted peak assigned to metallic Ag (9.6%) in the higher eV region than that of the other samples, as well as the Ag (III) peak (17.3%). The related Ag oxidation states and fits are shown in Supplementary Figure S6. These data support an increase in the formation of metallic Ag upon light exposure to Ag salts, as previously reported (Lou et al., 2011). Given this evidence, we suggest that in the absence of the chelating activity of SDF-ModP, the exposure of SDF-treated HAp to light results in a significant increase in metallic Ag and its respective oxides.

High-resolution O 1*s* spectra of SDF- and SDF+SDF-ModP-treated dentin samples revealed two primary oxygen peaks at 530.9/531.1 eV and 532.0/532.4 eV (Figures 4C,D). The lower-binding-energy pair (530.9/531.1 eV) corresponds primarily to phosphate-related oxygen (P–O) in the hydroxyapatite lattice, which commonly appears near ∼531 eV in Ca–P systems. An overlapping contribution from structural O–H in hydroxyapatite cannot be excluded, given the known proximity of the P–O and O–H components in the 531–533 eV window. The higher-binding-energy pair (532.0/532.4 eV) represents a combination of structural hydroxyl oxygen in hydroxyapatite together with organic oxygen from the dentin matrix (collagen carbonyl/C–O) and physisorbed/chemisorbed water from the aqueous environment, all of which typically appear within ∼531.5–533 eV (Ong et al., 1994; Schreckenbach et al., 1999; Zilm et al., 2016). The assignment of O 1*s* components in SDF-treated dentin is complicated by the coexistence of hydroxyapatite, collagen, aqueous adsorbates, and silver compounds. Silver-containing species generated by SDF treatment (Ag_2_O, AgO, or silver phosphates) may contribute to the 528–532 eV region (Bielmann et al., 2002; Kaspar et al., 2010). We emphasize the hydroxyapatite/organic/water interpretation for the 531–533 eV envelope while acknowledging the potential silver oxide overlap.

Additionally, the survey spectra of the samples revealed no F signal (Figures 3B,C). Instead, we observed Cl signals in both the SDF and SDF+SDF-ModP-treated samples, supporting the formation of AgCl, as reported previously (Mei et al., 2017; Mei et al., 2018). High-resolution XPS spectra of the Cl 2*p* region (Supplementary Figures S7A,B) revealed doublet-fitted peaks at almost identical locations for both SDF-and SDF+SDF-ModP-treated samples, around 199.1 eV for 2*p*_1/2_ and 197.4 eV for 2*p*_3/2_, values that are in good agreement with the reported data for AgCl (Wang et al., 2012). The presence of Ag_3_PO_4_ was expected as the reaction product of SDF with hydroxyapatite, but the signal originating from P 2*p* was observed at a very low intensity and could not be distinguished from the background noise. It is suspected that during incubation in SBF, silver phosphate was replaced with silver chloride over time (Mei et al., 2017).

### 3.3 Time-dependent analysis of color change

**FIGURE 6:**
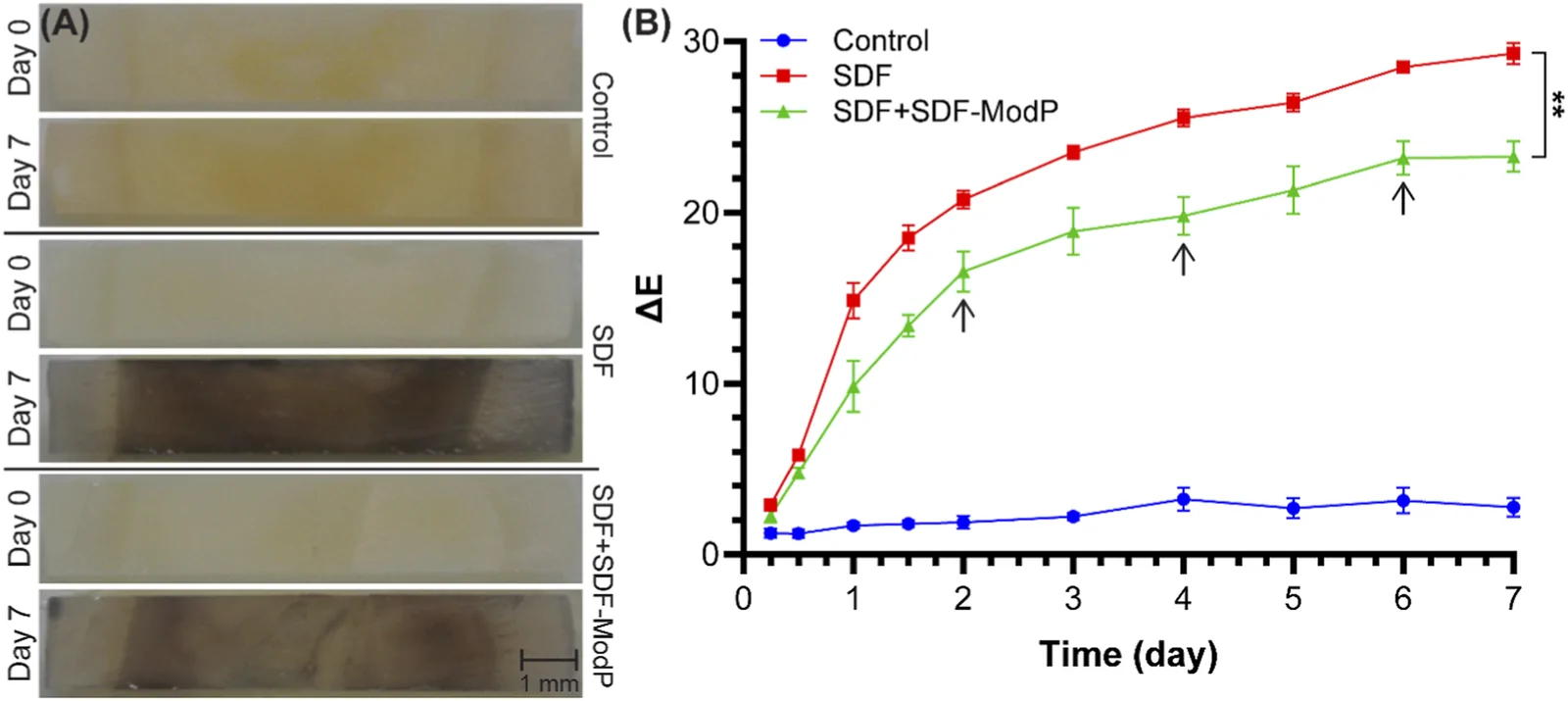
**(A)** Representative images of human dentin tissue slabs at baseline (Day 0) and after 1 week (Day 7) of incubation in SBF at 37°C, showing progressive staining in SDF-treated samples and reduced staining in SDF+SDF-ModP-treated samples; and **(B)** time course of color change (ΔE) quantified using CIELAB color space analysis. Arrows indicate timepoints of SDF-ModP reapplication (48 h intervals). Data represent mean ± SEM (n = 5 samples per group). **p < 0.001 by one-way ANOVA with Tukey’s *post hoc* test comparing SDF vs. SDF+SDF-ModP at Day 7.

Next, we examined whether SDF-ModP also affects the stain progression associated with SDF treatment by observing the change in the distribution of silver compounds with the use of SDF-ModP. We evaluated three groups of human dentin tissue slabs (Figure 6): untreated, SDF alone, and one treated with SDF-ModP following SDF application. All samples were incubated in SBF at 37 °C in the dark. The samples were exposed to light only when images were captured. SDF-ModP was reapplied following the same method every 48 h, and this event is marked with arrows (Figure 6B).

A negligible color change was observed in the untreated control group using the CIELAB space. Both SDF-treated groups exhibited varying degrees of staining, but the SDF+SDF-ModP samples showed a noticeably slower rate of color development than the SDF-only group. The captured images of the three sample groups were converted to the L*a*b* space, and the apparent change in the ΔE value was calculated. As illustrated in Figure 6B, it is evident that a noticeable divergence between the SDF-only and SDF+SDF-ModP-treated samples emerged after only 1 day of application. The SDF-only group showed the greatest color change over time, resulting in black staining across most of the dentin surface (Figure 6A). In contrast, the SDF+SDF-ModP group exhibited a more gradual color change, consistent with reduced aggregation of silver compounds and slower surface oxidation.

The application of silver diamine fluoride to tooth dentin structures initiates a sequence of reactions, starting with the release of silver ions from the SDF ion complex. Some of these silver ions react with hydroxyapatite to form silver phosphate and silver oxides, whereas others are reduced by light exposure or structural proteins such as the extracellular matrix, collagen, and SIBLING proteins (osteopontin, bone sialoprotein, dentin matrix protein 1, dentin sialophosphoprotein, and matrix extracellular phosphoglycoprotein (von Marschall and Fisher, 2008)) to form metallic silver, which subsequently forms a complex with proteins (Mei et al., 2013; Mei et al., 2017; Li et al., 2019; Hunwin et al., 2024). Moreover, some silver ions form silver halides, particularly silver chloride, by reacting with readily available chloride ions in saliva (Shah et al., 2018; Hunwin et al., 2024). In addition, in the presence of hydrogen sulfide in the oral environment, silver ions can react to form silver sulfide (Ag_2_S), a highly insoluble black compound that further contributes to the characteristic black staining following SDF treatment (Mei et al., 2018; Li et al., 2019; Toopchi et al., 2021). However, silver ions are primarily replaced by silver chloride and silver oxide, and a small portion is converted to silver sulfide along with the reduction of silver ions to metallic silver. The majority of the formed silver compounds are responsible for the formation of black stains and tooth color changes after SDF application (Mei et al., 2018; Sayed et al., 2020). The staining has been recognized as an impediment to wider use of SDF due to the esthetic stigma in each smile of the treated dentition. The SDF+SDF-ModP-treated samples exhibited a slower color change and lower overall discoloration compared to SDF-only samples in a time-based evaluation. This suggests that the peptide’s effect on silver compound behavior extends beyond chemical alteration, offering improved esthetic benefits. With increased proportion of Ag(I) species including AgCl, this may confer additional benefit, given that AgCl is recognized as one of the compounds most associated with SDF’s antibacterial reservoir function. We hypothesize that these effects may be driven by electrostatic features within the SDF-ModP sequence, an area which may need further investigations. The presence of electronegative surface domains likely facilitates interactions with silver cations, delaying oxidation and influencing compound distribution. These unique properties present an opportunity to design new, targeted peptide sequences for further optimization.

From a mechanistic perspective, our spectroscopic data support a sequential reaction model: (1) initial release of Ag^+^ ions from the SDF complex, (2) rapid precipitation of AgCl when chloride ions are present (e.g., in saliva or SBF), (3) partial oxidation of remaining silver species to Ag_2_O and AgO through reaction with dissolved oxygen and oxidizing agents in the tissue microenvironment, (4) potential formation of silver-protein and silver-phosphate coordination complexes at biointerfaces and (5) reduction of silver compounds to metallic silver over time through reduction reactions with the extracellular matrix, collagen, and SIBLING proteins in the instances of biominerals and with light exposure. The SDF-ModP peptide appears to intercede in this cascade, likely by coordinating Ag^+^ ions through its electronegative glutamic acid residues and modulating the kinetics of steps 2–5. The resulting altered distribution of silver compounds, but maintained AgCl formation and modified oxide ratios, can account for the reduced surface staining observed in the SDF-ModP-treated samples.

Our findings demonstrate that SDF-ModP modulates silver chemistry in SDF-treated dentin by shifting the equilibrium away from the rapid formation of metallic silver and silver oxides. This controlled interaction slows the visible stain development, and such a distribution may expand the effective antimicrobial depth of SDF-treated human dentin samples. These results highlight the potential of SDF-modulating peptides, such as SDF-ModP, to regulate silver compound formation, reduce black stain formation and esthetic drawbacks, and potentially improve the clinical performance of SDF treatments.

## 4 Conclusion

FDA-approved noninvasive SDF treatment for arresting early childhood dental caries continues to face significant drawbacks due to irreversible black tissue staining. Despite its success, our understanding of the underlying chemistry governing silver speciation at the SDF-dentin interface especially in carious dentin, remains limited. In this study, we explored a peptide modulator that interacts with the silver compounds of the SDF at the dentin-interface and shifts the silver compound formation from staining to preserving clinically beneficial silver chloride. We investigated the change in chemical states of silver content of the SDF with and without the presence of the peptide modulator (SDF-ModP). Using Raman micro-spectroscopy and X-ray photoelectron spectroscopy, the formation of metallic silver, silver oxides (Ag_2_O and AgO), and silver chloride compounds was confirmed following SDF treatment. SDF-ModP application shifted this distribution by reducing metallic silver and Ag(III)-associated AgO formation while increasing the proportion of Ag(I) species including AgCl. AgCl is recognized as one of the compounds most associated with SDF’s antibacterial reservoir function. One visible outcome of this modulation in silver chemistry was a noticeable reduction in the rate and extent of dark staining over the 7 days of observation. We hypothesize that the presence of electronegative surface domains in the peptide modulator likely facilitates interactions with silver cations, delaying oxidation and influencing compound distribution. These unique properties present an opportunity to design targeted peptides for diverse selective interactions.

Overall, this work demonstrates that peptide-enabled modulation of silver chemistry can enhance functionality and therapeutic efficacy, while reducing the adverse stain formation of SDF treatment. Future studies should continue to investigate the interaction mechanisms, ultimately guiding the development of next-generation, bioinspired materials and delivery systems for minimally invasive dental care. This peptide-modulated silver chemistry could be extended to other metal-based therapies where precise control of metal ion speciation is desired. The specific interactions of the peptides with metal ions may facilitate reduction or stabilization and extend the impact of targeted delivery systems and advanced materials for therapeutic applications.

## Data Availability

The original contributions presented in the study are included in the article/Supplementary Material, further inquiries can be directed to the corresponding author.
