# Supplementary material for: Modulating silver diamine fluoride chemical derivatives by solid-binding peptides
Source: Front Bioeng Biotechnol. 2026 Aug 10;14:1896633. doi: 10.3389/fbioe.2026.1896633 (PMC13500563; doi:10.3389/fbioe.2026.1896633)
Supplement: Supplementary file 1 [file DataSheet1.pdf]

Modulating Silver Diamine Fluoride Chemical Derivatives by Solid-Binding Peptides

Supplementary Material

1 Supplementary Figures and Tables

1.1 Supplementary Figures

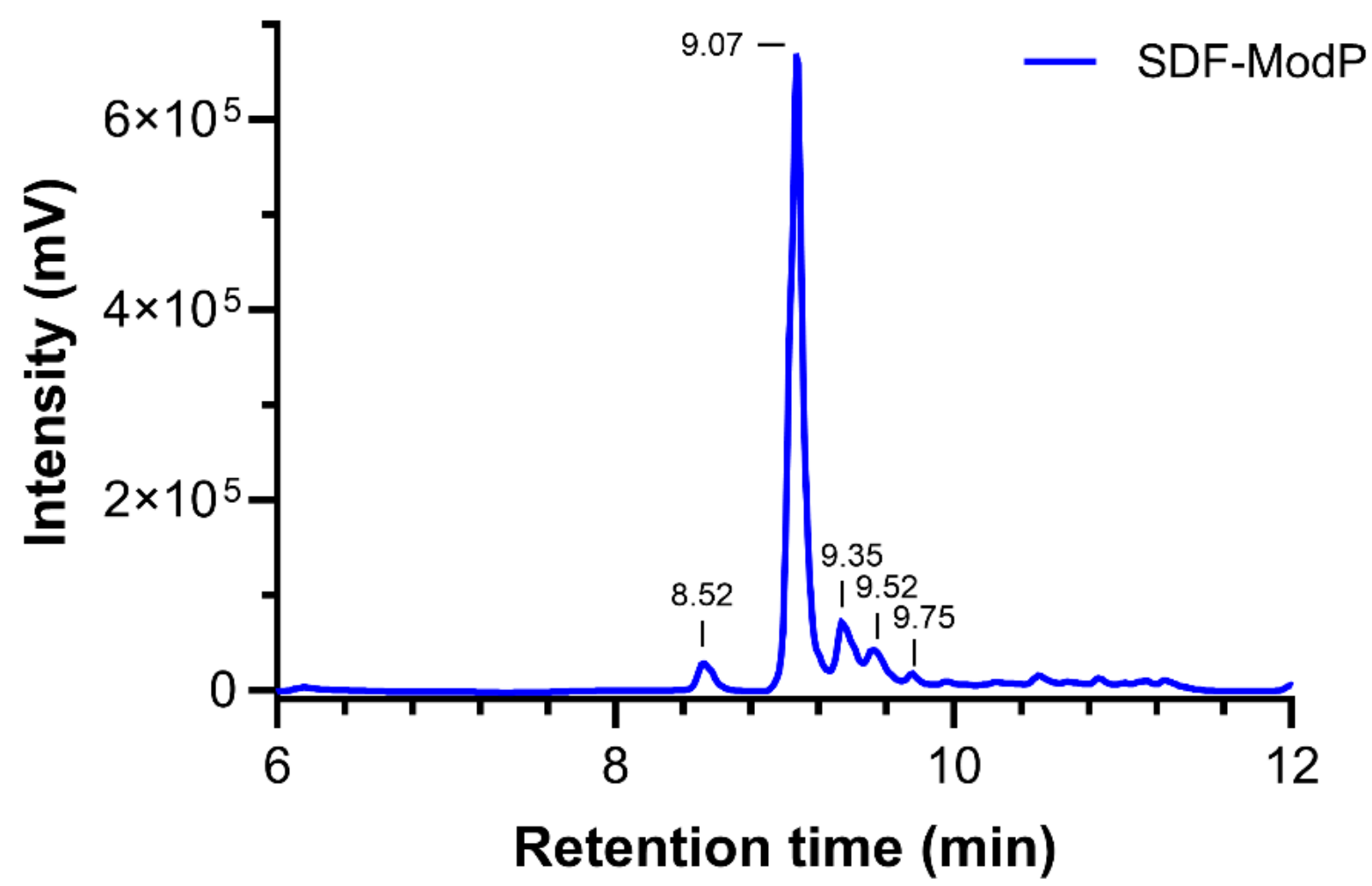

Supplementary Figure S1. HPLC chromatogram of SDF-ModP.

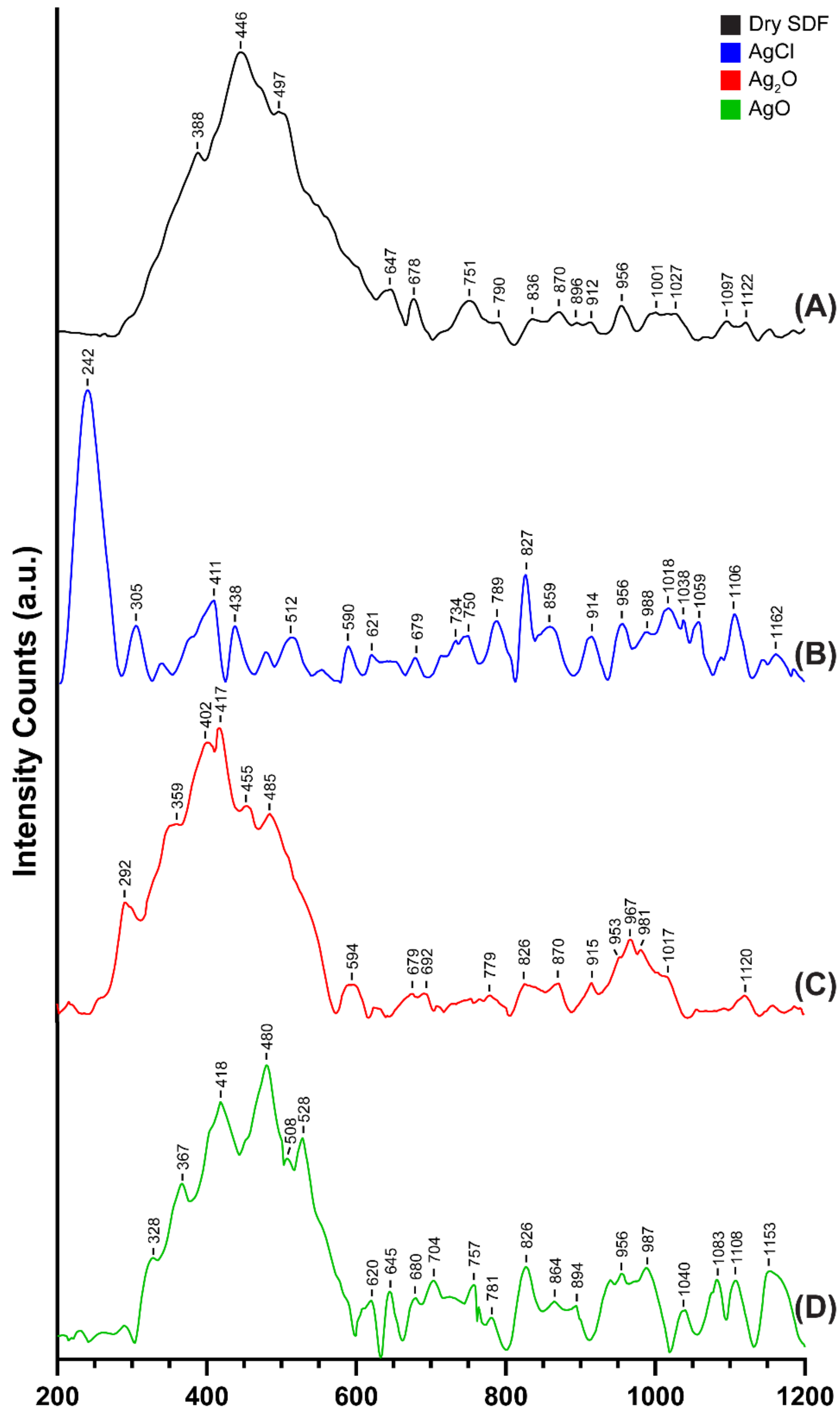

Supplementary Figure S2. Raman spectra of **(A)** dry SDF, **(B)** AgCl, **(C)** Ag_2O, and **(D)** AgO.

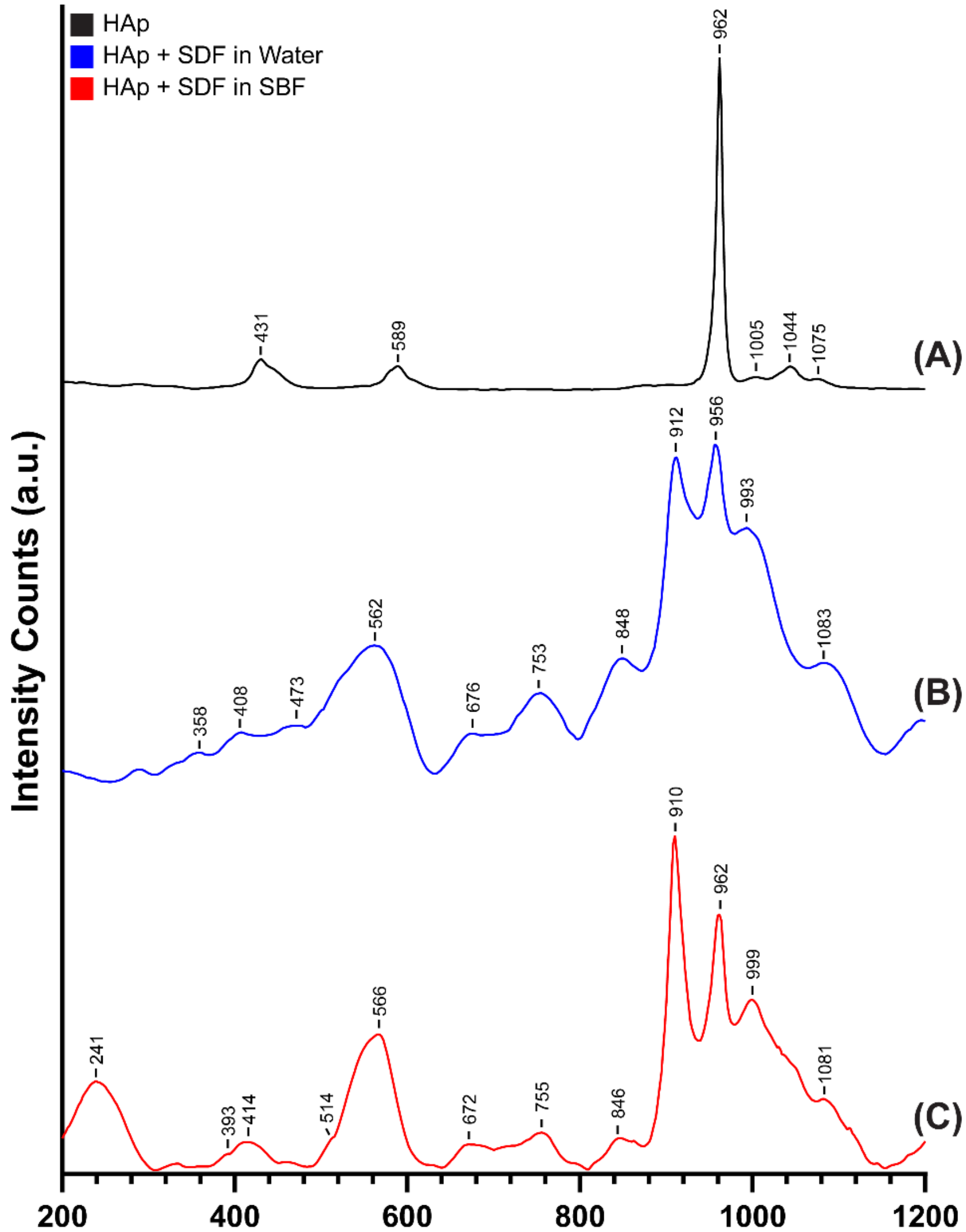

Supplementary Figure S3. Raman spectra of **(A)** HAp, **(B)** SDF-treated HAp incubated in water, and **(C)** SDF-treated HAp incubated in SBF.

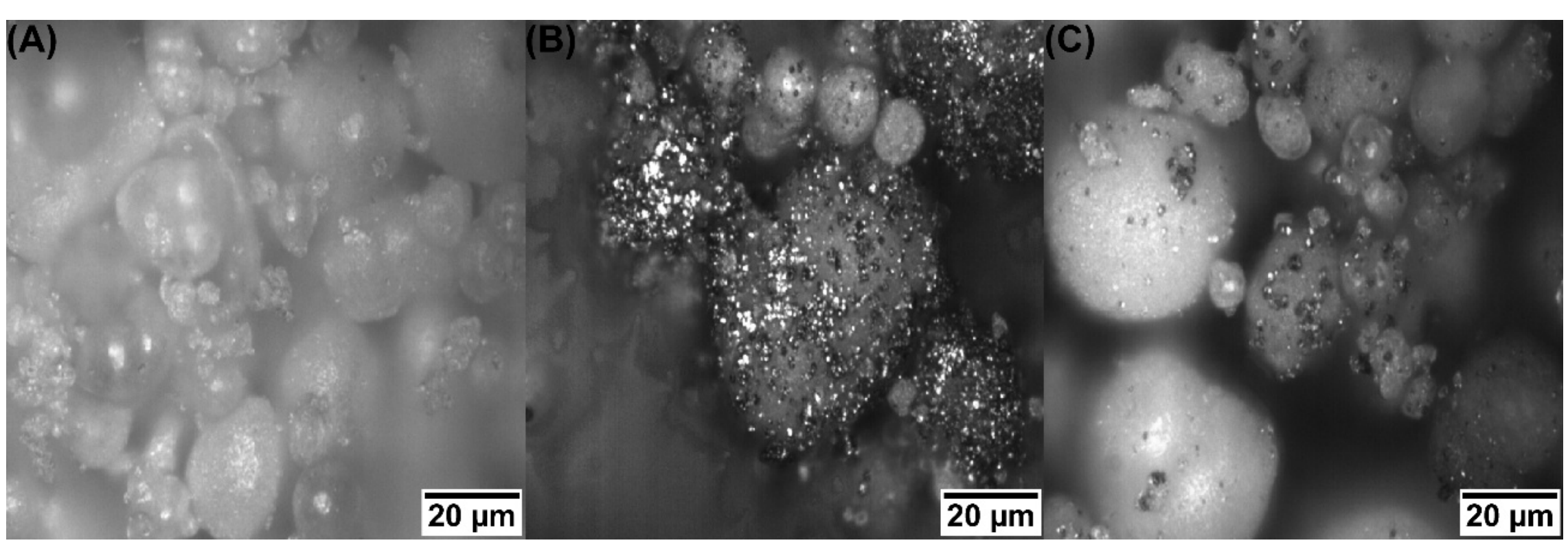

Supplementary Figure S4. Confocal microscopy bright-field images of **(A)** hydroxyapatite (HAp), **(B)** SDF-treated HAp in water, and **(C)** SDF-treated HAp in simulated body fluid (SBF).

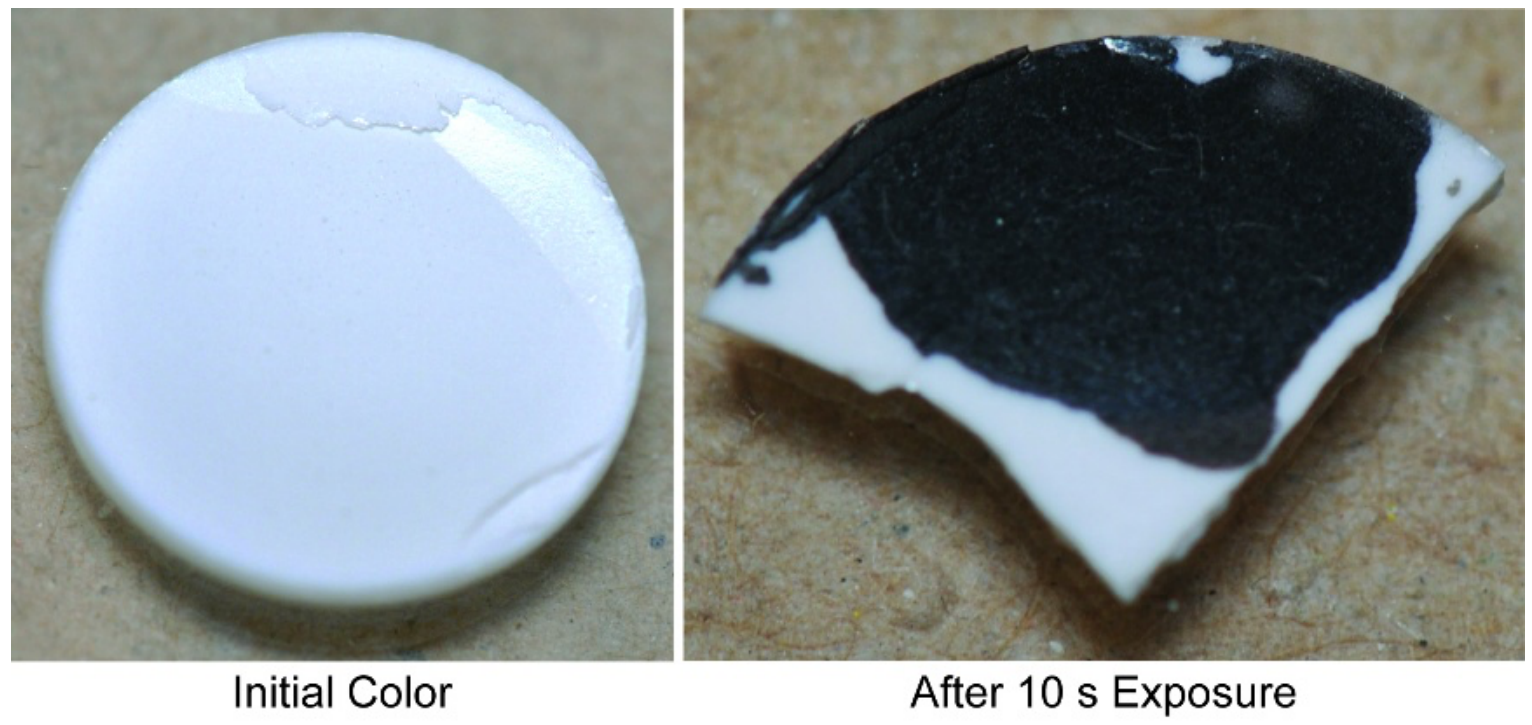

Supplementary Figure S5. SDF-treated HAp disk before and after 10 seconds of 550 mW/cm^2 light treatment.

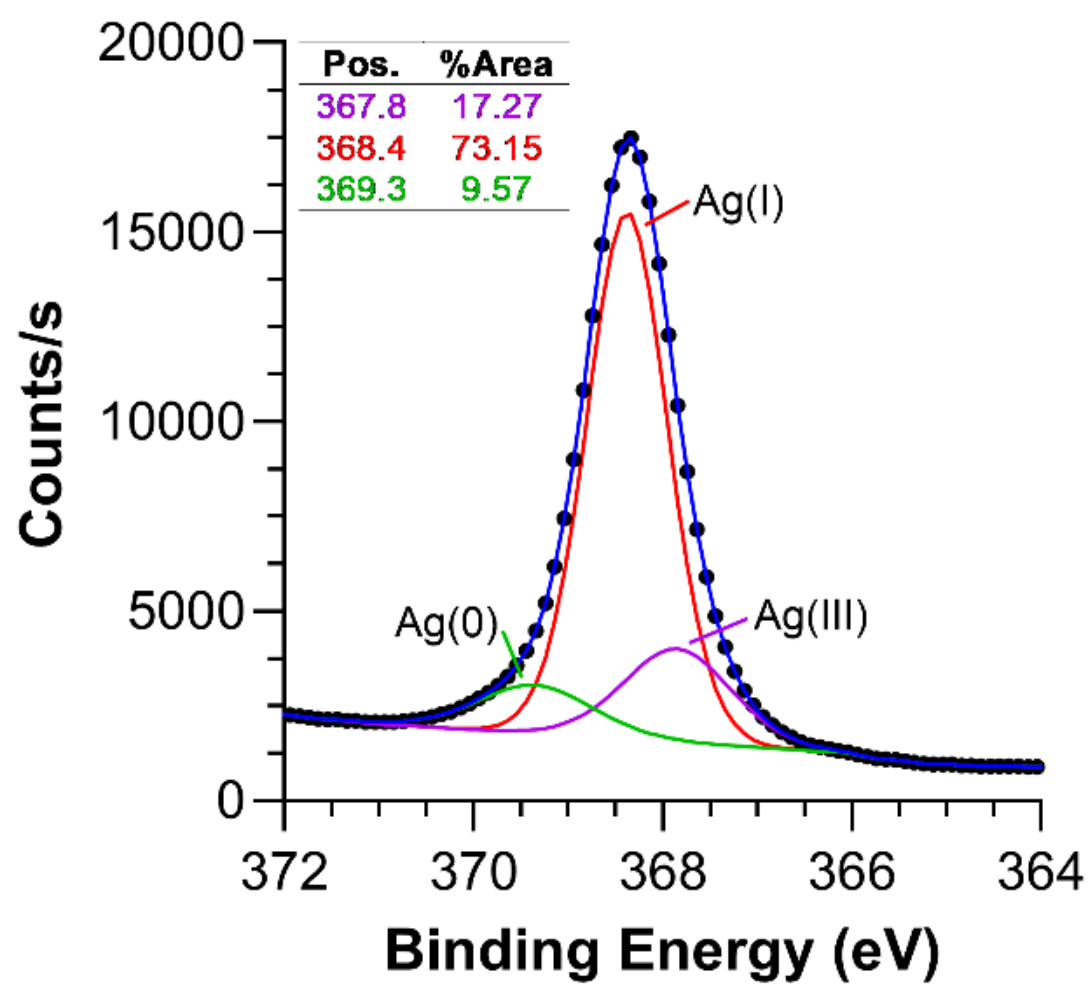

Supplementary Figure S6. High-resolution XPS data illustrating the Ag 3$d_{5/2}$ region for SDF-treated and light-exposed HAp.

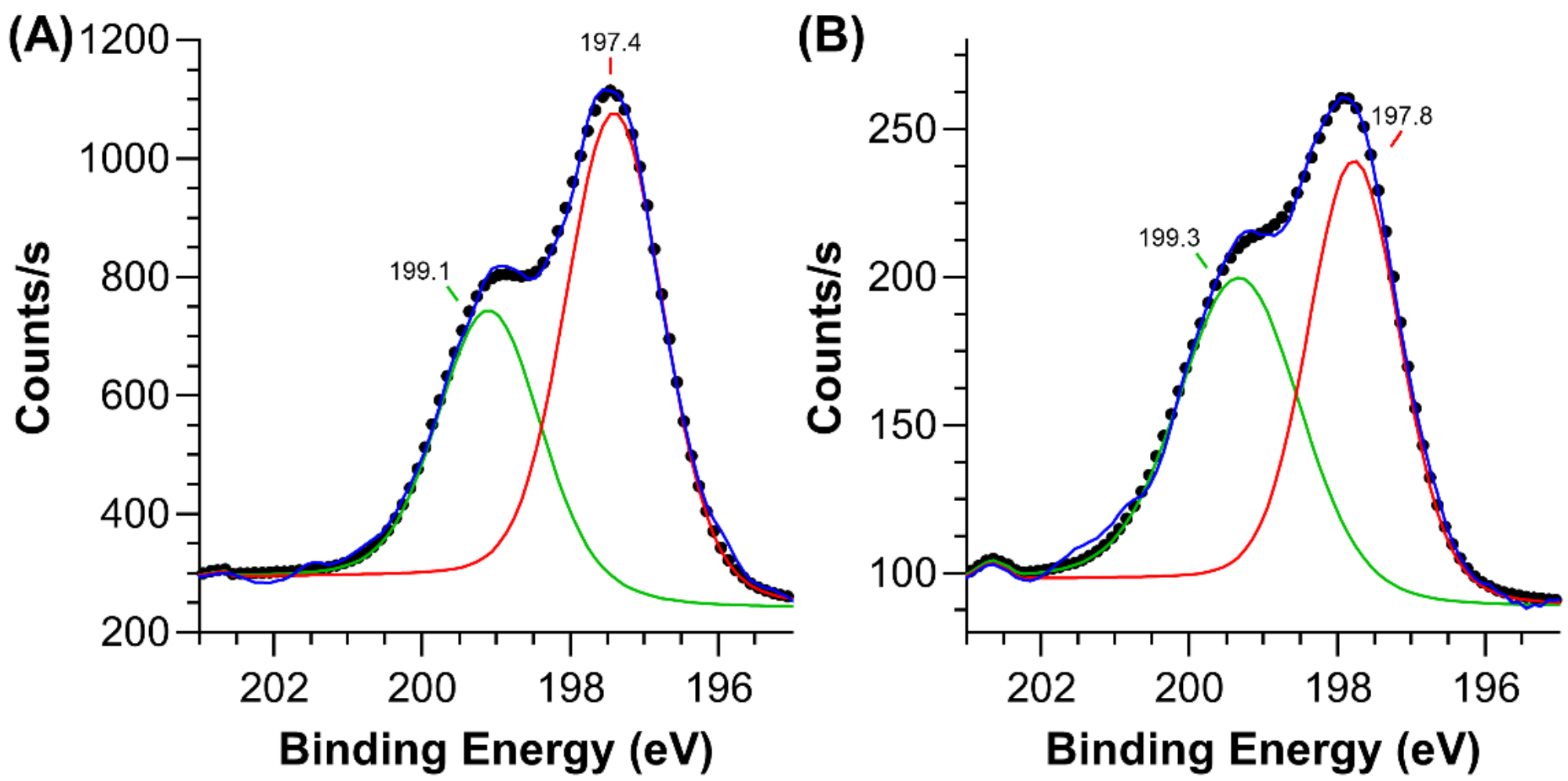

Supplementary Figure S7. High-resolution XPS data illustrating the Cl 2*p* region for **(A)** SDF-treated and **(B)** SDF+SDF-ModP-treated dentin.

1.2 Supplementary Tables

Supplementary Table S1. Reagents, amounts for preparing 1,000 mL of SBF, final concentration of ions, and concentration in blood plasma (Oyane et al., 2003; ISO, 2014; Srisomboon et al., 2022).

Reagent	Amount	Conc. (mM)	Ion	Conc. (mM)	Blood Plasma (mM)
NaCl	8.035 g	137.5	Na^{+}	142.7	142.0
$NaHCO_3$	0.355 g	4.2	K^{+}	5.0	5.0
KCl	0.225 g	3.0	Mg^{+2}	1.5	1.5
$K_2HPO_4.3H_2O$	0.231 g	1.0	Ca^{+2}	2.5	2.5
$MgCl_2.6H_2O$	0.311 g	1.5	Cl^{-}	185.5	103.0
HCl	40 mL	40.0	HCO^{-3}	4.2	27.0
$CaCl_2$	0.278 g	2.5	HPO_4^{-2}	1.0	1.0
Na_2SO_4	0.072 g	0.5	SO_4^{-2}	0.5	0.5
Tris	6.118 g	50.5			

References

ISO (2014). *Implants for Surgery: In Vitro Evaluation for Apatite-forming Ability of Implant Materials (ISO 23317:2014).* ISO Copyright Office.

Oyane, A., Kim, H.M., Furuya, T., Kokubo, T., Miyazaki, T., and Nakamura, T. (2003). Preparation and assessment of revised simulated body fluids. *Journal of Biomedical Materials Research Part A* 65(2)**,** 188–195. doi: 10.1002/jbm.a.10482.

Srisomboon, S., Kettratad, M., Stray, A., Pakawanit, P., Rojviriya, C., Patntirapong, S., et al. (2022). Effects of Silver Diamine Nitrate and Silver Diamine Fluoride on Dentin Remineralization and Cytotoxicity to Dental Pulp Cells: An In Vitro Study. *Journal of Functional Biomaterials* 13(1)**,** 16. doi: 10.3390/jfb13010016.
